# Proteomics in Liver Transplantation: A Systematic Review

**DOI:** 10.3389/fimmu.2021.672829

**Published:** 2021-07-26

**Authors:** Victor López-López, Fernando Pérez-Sánz, Carlos de Torre-Minguela, Josefa Marco-Abenza, Ricardo Robles-Campos, Francisco Sánchez-Bueno, José A. Pons, Pablo Ramírez, Alberto Baroja-Mazo

**Affiliations:** ^1^ Department of Surgery, Hospital Clínico Universitario Virgen de la Arrixaca, Murcia, Spain; ^2^ Digestive and Endocrine Surgery and Transplantation of Abdominal Organs, Biomedical Research Institute of Murcia (IMIB-Arrixaca), Murcia, Spain; ^3^ Biomedical Informatic and Bioinformatic Platform, Biomedical Research Institute of Murcia (IMIB-Arrixaca), Murcia, Spain; ^4^ School of Nursing, Universidad de Murcia, Murcia, Spain; ^5^ Department of Gastroenterology, Unit of Hepatology, Hospital Clínico Universitario Virgen de la Arrixaca, Murcia, Spain

**Keywords:** rejection, tolerance, mass spectrometry, ischemia – reperfusion, PRISMA

## Abstract

**Background:**

Although proteomics has been employed in the study of several models of liver injury, proteomic methods have only recently been applied not only to biomarker discovery and validation but also to improve understanding of the molecular mechanisms involved in transplantation.

**Methods:**

The study was conducted following the Preferred Reporting Items for Systematic Reviews and Meta-Analyses (PRISMA) methodology and the guidelines for performing systematic literature reviews in bioinformatics (BiSLR). The PubMed, ScienceDirect, and Scopus databases were searched for publications through April 2020. Proteomics studies designed to understand liver transplant outcomes, including ischemia-reperfusion injury (IRI), rejection, or operational tolerance in human or rat samples that applied methodologies for differential expression analysis were considered.

**Results:**

The analysis included 22 studies after application of the inclusion and exclusion criteria. Among the 497 proteins annotated, 68 were shared between species and 10 were shared between sample sources. Among the types of studies analyzed, IRI and rejection shared a higher number of proteins. The most enriched pathway for liver biopsy samples, IRI, and rejection was metabolism, compared to cytokine-cytokine receptor interactions for tolerance.

**Conclusions:**

Proteomics is a promising technique to detect large numbers of proteins. However, our study shows that several technical issues such as the identification of proteoforms or the dynamic range of protein concentration in clinical samples hinder the successful identification of biomarkers in liver transplantation. In addition, there is a need to minimize the experimental variability between studies, increase the sample size and remove high-abundance plasma proteins.

## Introduction

Liver transplantation (LT) for the treatment of end-stage liver diseases remains debatable regarding graft viability, such as acute rejection, ischemia-reperfusion injury (IRI), or primary graft dysfunction. To predict and prevent dysfunction and graft rejection, researchers have tried to identify biomarkers that can predict which grafts are more likely to suffer these complications (1). Many studies have focused on DNA polymorphisms, biopsy RNA expression or donor circulant-free DNA and microRNA for the prediction and diagnosis of several events related to transplantation (2–4). Although proteomics has been applied in the study of liver injury (5), proteomic methods have only recently started to been used for biomarker discovery but also to improving the understanding of the molecular mechanisms in transplantation (6). Proteomics is defined as the unbiased global analysis of the quantitative expression and identification of proteins in a cell, tissue, or organ. Instead of the classical approach in which a candidate gene or protein guides all analyses, protein profiling provides a powerful method to analyze the role of proteins in disease processes in an unbiased manner. Proteomics assays capture a large set of proteins, providing an approach to identify possible biomarkers and mediators and the advantage of elucidating the overall patterns of lesion-induced changes at the protein level (7). Moreover, functional proteomics provides a superior capacity over other techniques to identify modified proteins involved in multiple networks of living cells or body fluids (8). In addition, proteomics complements gene profiling because it represents the regulation of translation, post-translational modifications, protein kinetics, protein-protein interactions, and protein losses. Integrative approaches are expected to unify high-throughput genomics, proteomics and bioinformatics to develop what is known as “transplantomics” in the field of transplantation (9). The aim of proteomics is to unravel all underlying biological mechanisms to obtain information on the physiopathology of the alloimmune response, rejection, or IRI and, thus, to define new therapeutic targets (10). Studies have shown that the proteins involved in lipid and energy metabolism, redox signaling, and oxidative stress are affected during both the hot and cold ischemic phases of transplantation (11). Proteomic research can also be used to identify immunologic markers for operational tolerance in liver or kidney graft recipients. Regarding rejection, the availability of clinical biomarkers of immune activation may allow individualized patient management to avoid acute rejection events (11).

Here, we review the use of proteomics in LT including graft rejection, IRI, immune tolerance, and other transplant derived diseases. To do so, we analyzed different reported techniques and sample sources and compared human and animal studies to describe the principal relevant proteins overlapping among independent studies. Moreover, we highlight the limitations in the proteomic approaches of the different studies as one of the major challenge for the future of this research.

## Experimental Procedures

This systematic literature review (SLR) was carried out following the Preferred Reporting Items for Systematic Reviews and Meta-Analyses (PRISMA) checklist (12) and the guidelines for performing SLR in bioinformatics (BiSLR) (13).

### Search Strategy

We performed a systematic review of scientific publications that analyzed the proteomic profiles of liver transplant samples. All relevant articles were searched without date limits from the PubMed, Scopus, and ScienceDirect databases. The search terms included a combination of standardized index terms and plain language to cover the terms “liver transplantation”; “liver graft”; “liver transplant”; “liver allograft”; “proteomics”; and “proteome”. These keywords were defined by consensus among authors and customized for each database (**Table S1**). The search was completed in April 2020.

### Selection Criteria

The searches were limited to studies published in the English language using the standard limitations provided by the respective databases. To be eligible for screening, the studies had to meet the following criteria: (a) identification of LT proteomics biomarkers as the main objective, (b) including human or animal models, and (c) application of methodologies for differential expression analysis from proteome profiling. Studies lacking adequate information on the experimental design were not included. Abstracts, book chapters, and review papers were also not considered.

### Study Selection

To reduce the risk of bias (13), four researchers from different fields (bioinformatics, biology, proteomics, and medicine) independently reviewed the retrieved articles to assess their suitability for inclusion in this review. After removing duplicates using Mendeley reference management software, study selection was performed in four steps: (a) title screening, (b) abstract screening, (c) diagonal reading (focused on introduction, figures, tables, and conclusion); and (d) full-text reading (13). Only when more than half of the authors accepted inclusion based on steps (a) to (c), those studies were selected for the next step (d), where each reviewer independently evaluated the articles according to a scoring system and the following set of five questions (1): “does the article try to identify biomarkers related to liver transplant?” (2); “has the article generated proteome data?;” (3) “is the methodology based on differential expression analysis of proteome data?” (4); “does the article identify the experimental design, groups, and types or number of samples?”; and (5)”does the article properly report the list of potential biomarkers and the respective fold-change, *p-*value, or differential patterns observed (up-regulation or down-regulation) in the main text or supplementary material?”. For each question, scores were given following predefined criteria: 0 if the article does not meet any requirement of the question; 1 if it only partially meets the requirements; and 2 if it meets all the requirements. Articles with an average score equal to or higher than 6 among all evaluations were included.

### Analysis and Data Collection

Data were independently extracted by all investigators using a standardized form and disagreements were resolved by consensus. A narrative summary of the results was produced according to specific data subjects: (a) the article’s author and year of publication; (b) study characteristics, including the design, study model, study type, sample type, proteomic technology, data analysis methods, presence or absence of validation; and (c) differential expression patterns of potential biomarkers (up- or down-regulated). For each study, the reported proteins were extracted from tables, figures, text, or supplemental material. For evaluation purposes, the protein identifiers were converted to the official gene symbol nomenclature (14).

### Pathway Enrichment Design

Pathway and gene ontology (GO) enrichment analyses were performed for the differentially expressed proteins in the different study types (IRI, rejection, tolerance) or sample sources (plasma/serum or biopsies), using the corresponding functions of the limma package in the R statistics language (15).

These functions perform over-representation analyses by computing one-sided hypergeometric tests equivalent to Fisher’s exact tests. The *p*-values returned by these functions are not adjusted for multiple testing because the terms and pathways are often overlapping and standard methods of *p*-value adjustment may be very conservative. Therefore, caution should be exercised when interpreting the results. Only very small *p*-values should be considered.

### Risk of Bias Measurement

Due to the large heterogeneity in the study types, we adapted and answered 13 items of the Downs and Black checklist for a critical evaluation of the quality of the cited papers (**Table S2**) (3).

## Results

### Literature Search and Studies Descriptions

**Figure 1:**
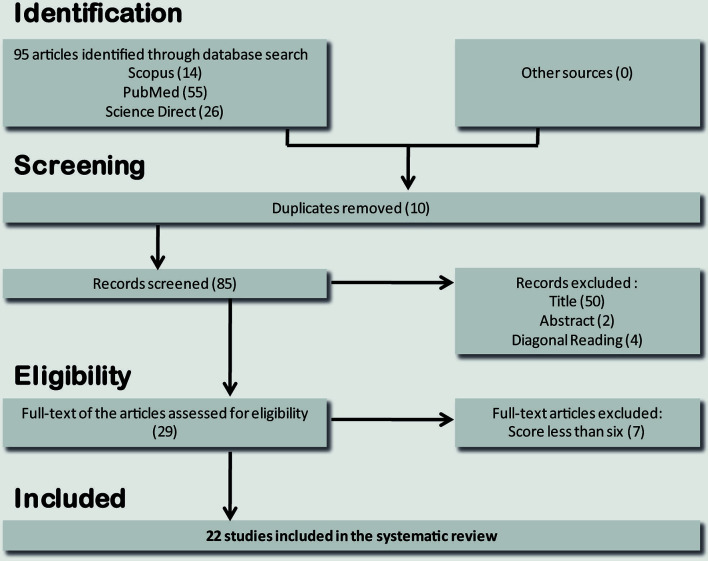
Flow diagram through the different phases of studies selection for systematic review.

A total of 95 references were collected from the PubMed, ScienceDirect, and Scopus databases. Duplicates were removed, resulting in 85 studies. After the screening phase, 56 studies were excluded. Among the remaining 29 studies, seven were excluded for average scores below 6. Finally, the SLR included 22 studies (**Figure 1**). Fifty percent of the studies were from Asia, 27% were from the US, and 18% were from Europe. Only one paper was from South America (16). The selected studies were published between 2004 and 2019, with 40% published in the last 5 years (**Table 1**).

**Table 1:** Principal characteristics of the publications selected for this SLR.

Author	Country	Year	Model	Type of sample	Type of study	Sample size*
Pan et al. (17)	Taiwan	2004	Rat	Serum	Tolerance	n.d.
Vascotto et al. (18)	Italy	2006	Human	Biopsy	IRI	9
Zhang et al. (19)	China	2007	Rat	Biopsy	Rejection	9
Svetlov et al. (20)	USA	2008	Rat	Biopsy	IRI	3
Cheng et al. (21)	China	2010	Rat	Biopsy	Rejection	n.d.
Wu et al. (22)	China	2010	Rat	Biopsy	Liver regeneration	15
Pan et al. (23)	Taiwan	2011	Rat	Serum	Tolerance	n.d.
Parviainen et al. (24)	Finland	2011	Human	Plasma	IRI	3
Levitsky et al. (25)	USA	2011	Human	Plasma	CKD	64
Massoud et al. (26)	USA	2011	Human	Serum	Rejection	8
Tiriveedhi et al. (27)	USA	2012	Rat	Biopsy	IRI	3
Kornasiewicz et al. (28)	Poland	2012	Human	Biopsy	Primary graft non-function	96
Wu et al. (29)	China	2012	Human	Biopsy	IRI	3
Levitsky et al. (30)	USA	2013	Human	Plasma	Tolerance	20
Wei et al. (31)	China	2015	Rat	Biopsy	Rejection	10
Toby et al. (32)	USA	2017	Human	PBMCs	Rejection	26
Wang et al. (33)	Taiwan	2018	Rat	Serum	Tolerance	5
Knecht et al. (16)	Argentine	2018	Rat	Biopsy	IRI	4
Jiang et al. (34)	China	2019	Human	Serum	Rejection	6
Coskun et al. (35)	Turkey	2019	Human	Preservation solution	IRI	23
Huang et al. (36)	China	2019	Human	Biopsy	IRI	13
Zhang et al. (37)	China	2019	Human	Serum	IRI	9

*total number of patients per trial or number of rats per group of study. n.d., not determined; IRI, ischemia-reperfusion injury; CDK, chronic kidney disease.

### Proteomics Strategies

Two-dimensional gel electrophoresis (2D gel)- based proteomics is the most widely used strategy described in selected publications (50%); however, shotgun proteomics has displaced this strategy, as this proteomic method has been applied most frequently in the last 5 years (62.5%). Only four of the publications used antibody arrays, two of which were in combination with 2D gel-based proteomics analyses (20, 33) (**Table 2**). The application of prefractionation techniques to reduce the high dynamic range in protein abundance is increasing, as we observed that their use has doubled in the last 5 years (62.5% *vs*.21.4%). Protein identification was carried out mainly by tandem mass spectrometry (MS/MS) (75%), but only half of the selected publications performed validation, principally by western blot (80%). The protein quantification most used in 2D gel-based proteomics was silver staining (50%), while stable isotope labeling of peptides with isobaric tags for relative and absolute quantitation (iTRAQ) method (62%) was the most often used method for shotgun proteomics (**Table 2**). In addition, two publications carried out fluorescent multiplexing technology (25, 30). The median number of selected proteins differentially expressed per study was 37, ranging from 7 (33) to 197 (35) (**Table 2**).

**Table 2:** Proteomics technology used along the different selected publications in this SLR.

Author	Year	Type of sample	Proteomics technology	Quantification method (range of isoelectric point)	Prefractionation	Identification		Number of selected proteins	Validation
						Mass spectrometer	Method		
Pan et al.	2004	Serum	2D gel based-proteomics	Coomassie Brilliant Blue (3-10)	NO	MALDI-TOF MS	PMF	13	–
Vascotto et al.	2006	Biopsy	2D gel based-proteomics	Silver (3-10)	NO	MALDI-TOF MS	PMF	36	–
Svetlov et al.	2006	Biopsy	Antibody array/SDS-PAGE & LC-MS/MS	Antibodies fluorescence	Cationanion exchange​ chromatography and SDS-PAGE	LC-ESI-MS/MS	MS/MS	17	WB
Zhang et al.	2007	Biopsy	2Dgel based-proteomics	DIGE (3-10 NL)	NO	MALDI-TOF MS	PMF	16	WB
Cheng et al.	2010	Biopsy	2Dgel based-proteomics	Silver (3-10 NL)	NO	MALDI-TOF/TOF MS	MS/MS	18	WB
Wu et al.	2010	Biopsy	2Dgel based-proteomics	Silver (3-10)	NO	MALDI-TOF/TOF MS	MS/MS	9	WB
Pan et al.	2011	Serum	2Dgel based-proteomics	DIGE (4-7)	NO	MALDI-TOF MS & LC-ESI-MS/MS	PMF and MS/MS	19	WB
Parviainen et al.	2011	Plasma	Shotgun Proteomics	iTRAQ	Agilent Multiple Affinity Removal LC Column-Human 6	LC-ESI-MS/MS	MS/MS	72	–
Levitsky et al.	2011	Plasma	Luminex Bead Technology	Antibodies fluorescence	NO	–	–	22	–
Massoud et al.	2011	Serum	Shotgun Proteomics	iTRAQ	Multiple Affinity Removal Column (6) + strong cation exchange column fractionation	LC-ESI-MS/MS	MS/MS	41	ELISA
Tiriveedhi et al	2012	Biopsy	2Dgel based-proteomics	DIGE (3-10 NL)	NO	LC-ESI-MS	MS/MS	106	–
Kornasiewicz et al.	2012	Biopsy	2Dgel based-proteomics	Silver (3-10)	NO	LC-ESI-MS	PMF	21	–
Wu *et al.*	2013	Biopsy	2Dgel based-proteomics	DIGE (3-10)	NO	MALDI-TOF/TOF MS	MS/MS	34	WB
Levitsky et al.	2013	Plasma	Luminex Bead Technology	Antibodies fluorescence	NO	–	–	12	–
Wei et al.	2015	Biopsy	Shotgun Proteomics	iTRAQ	Strong cation exchange liquid chromatography (SCX)	MALDI-TOF/TOF MS	MS/MS	57	WB
Toby et al.	2017	PBMCs	TOP-Down proteomics	Label free	Gel-eluted liquid fraction entrapment electrophoresis (GELFrEE). Fraction 0-30 kDa	LC-ESI-MS/MS	MS/MS	51	Not required
Wang et al.	2018	Serum	2Dgel based-proteomics/Antibody array	Silver (4-7)	NO	MALDI-TOF MS	PMF	7	WB
Knect et al.	2018	Biopsy	2Dgel based-proteomics	Coomassie Brilliant Blue (3-10)	NO	MALDI TOF/TOF MS	MS/MS	23	–
Jiang et al.	2019	Serum	Shotgun Proteomics	iTRAQ	ProteoMiner protein enrichment	LC-ESI-MS/MS	MS/MS	10	ELISA
Coskun *et al.*	2019	Preservation solution	Shotgun Proteomics	Label free	Immunodepletion Hu14 column	LC-ESI-MS/MS	MS/MS	197	–
Huang et al.	2019	Biopsy	Shotgun Proteomics	TMT tags	NO	LC-ESI-MS/MS	MS/MS	10	–
Zhang et al.	2019	Serum	Shotgun Proteomics	iTRAQ	Phase reverse fractionation	LC-ESI-MS/MS	MS/MS	22	–

SDS-PAGE, Sodium dodecyl sulfate-polyacrylamide gel electrophoresis; 2D gel, Two-dimension gel electrophoresis; DIGE, Differential in gel electrophoresis; iTRAQ, Isobaric tags for relative and absolute quantitation; TMT tag, Tandem mass tag system; MALDI, Matrix-assisted laser desorption/ionization; TOF, Time-of-flight; LC-, Liquid chromatography; ESI, Electrospray ionization; PMF, Peptide mass fingerprinting; MS/MS, Tandem mass spectrometry; WB, Western blot; ELISA, Enzyme-linked immunoSorbent assay.

### Sample Sources and Study Types

Half of the publications were conducted as clinical trials with human samples, whereas the other half used rats as an animal model (**Table 1**). The sample size was highly variable for human studies, with a mean of 23 patients per trial, with a range from 3 to 96 patients (**Table 1**). The mean number of rats per analysis group was 7, with a minimum of 3 and a maximum of 15 (**Table 1**). Likewise, most of the proteomics were performed with liver biopsies (50%), followed by serum or plasma (41%) (**Table 1**). Only one study each used peripheral blood mononuclear cells (32) or preservation solution (35) as sample source. Most of the selected publications focused on liver IRI (41%), followed by acute liver graft rejection (27.3%) and immunological LT tolerance (18.2%). Only one paper each analyzed three other scenarios such as primary graft non-function (28), chronic kidney disease (CKD) associated with LT (25) or liver regeneration after living-donor LT (22) (**Table 1**). The IRI studies were quite variable. Among human samples, two publications analyzed samples only after reperfusion. Zhang et al. (37) compared healthy volunteers to liver transplant patients 1 to 7 days after reperfusion, while Parvanien et al. (24) took samples from the portal vein, the hepatic vein, and the radial artery, and compared changes among the different types of blood. On the other hand, Coskun et al. (35) analyzed cold ischemia using organ preservation solution as a sample source. Three other studies recollected both ischemia and reperfusion samples and compared them to control (pre-ischemia) samples and even between them (18, 29, 36). When rats were used as a model, Knecht et al. (16) carried out *ex vivo* cold storage and normothermic reperfusion, whereas the other 2 publications performed a model of *in vivo* selective warm ischemia using vascular clamping followed by reperfusion, such in normal (20) as in steatotic rats (27).

Only one study on rejection analyzed chronic rejection (31), whereas the rest were focused on acute cellular rejection, of which, two were carried out in rats (19, 21) and three with human samples (26, 32, 34), although Massoud et al. (26) enrolled hepatitis C virus -positive patients in their trial.

In contrast, spontaneous tolerance in a rat model of LT was the main model used in the tolerance selected publications (17, 23, 33).

### Commonly Identified Proteins Among Independent Studies

**Figure 2:**
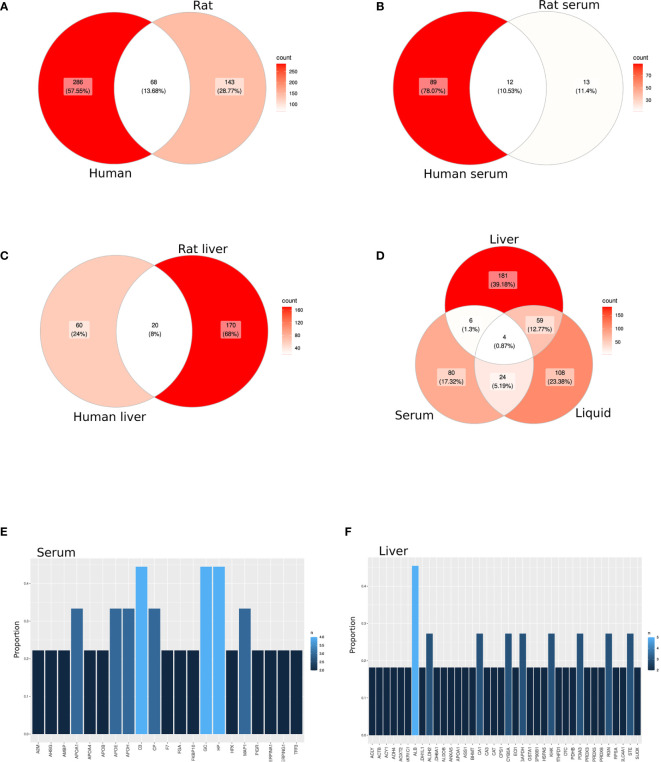
Candidate protein biomarkers from different sample source. **(A–D)** Venn diagram among all selected studies in a cross-species **(A–C)** or cross-sample source **(D)** analysis. **(E, F)** Bar graph representing the most shared proteins among all selected studies in serum/plasma **(E)** or liver biopsies **(F)**.

A total of 497 proteins detected by proteomics were identified by the selected studies (354 and 211 from humans and rats, respectively) (**Table S3**). Of these, 68 proteins (13.68%) overlapped between species (**Figure 2A**; **Table S4A**). Among 114 proteins identified in serum or plasma (101 in human and 25 in rats), 12 (10.53%) overlapped between species (**Figure 2B**; **Table S4B**). Among 250 proteins detected in liver biopsy studies (80 in human and 190 in rat), 20 (8%) overlapped between species (**Figure 2C**; **Table S4C**). Almost half of the proteins annotated from serum or plasma (45.6%) were among the 150 most abundant proteins in plasma (38) (**Table S4D**).

Likewise, only 10 proteins (2.17%) overlapped between serum and tissue samples, including both animal and human samples (**Figure 2D**; **Table S4E**). However, 63 (13.64%) and 28 (6.1%) proteins overlapped between preservation solution and liver biopsies or serum, respectively (**Figure 2D**; **Tables S4F, G**). Moreover, four proteins (0.87%), including C-reactive protein (CRP), Glutathione S-transferase alpha 1 (GSTA1), Hemoglobin subunit beta (HBB) and Hemopexin, were found in all sample sources (**Table S4H**).

Among the selected studies based on serum or plasma, three proteins were found in 44.44% of the publications (Complement C3 (C3), GC vitamin D binding protein (GC) and Haptoglobin), whereas other five proteins [Apolipoprotein A1 (APOA1), Apolipoprotein E (APOE), Apolipoprotein H (APOH), Ceruloplasmin and Microtubule associated protein 1 (MAP1)] were found in one-third of the studies (**Figure 2E**; **Table S4I**). However, when liver biopsies were the sample source for proteomics, only albumin was found in almost half of the publications, followed by eight proteins differentially expressed in at least three publications (27.3%) (**Figure 2F**; **Table S4J**).

**Figure 3:**
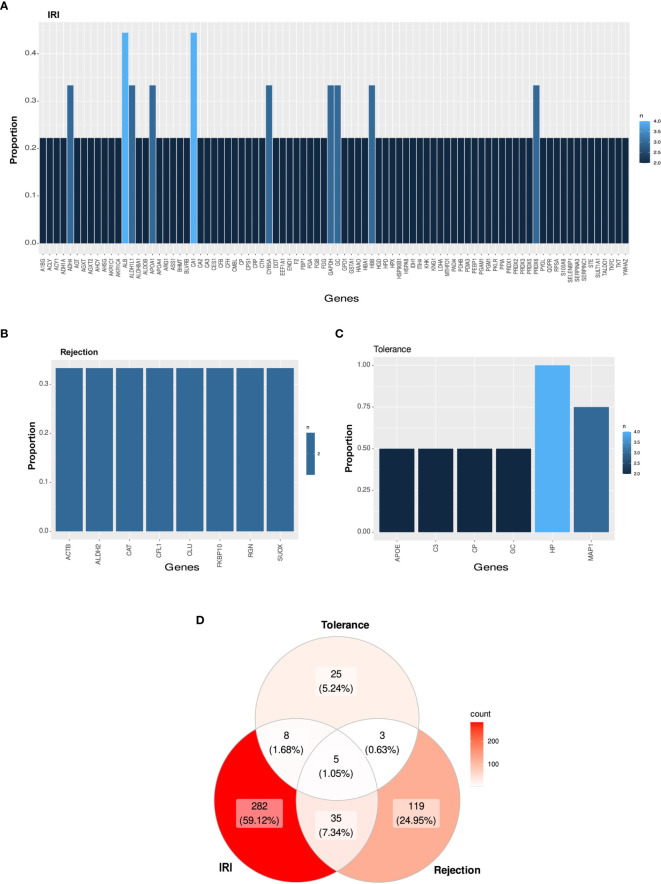
Candidate protein biomarkers from different types of study. **(A–C)** Bar graph representing the most shared proteins among all selected studies in IRI **(A)**, rejection **(B)** or tolerance **(C)**. **(D)** Venn diagram among all selected publications in cross-type of study analysis.

On the other hand, two proteins [albumin and carbonic anhydrase 1 (CA1)] were described as differentially expressed in four of the nine selected works related to IRI and eight proteins in three publications, whereas other 76 proteins were annotated in two studies (**Figure 3A**; **Table S4K**). Likewise, eight proteins were shared in one-third of the graft rejection studies [Actin beta (ACTB), Aldehyde dehydrogenase 2 family member (ALDH2), Catalase, Cofilin 1 (CFL1), Clustering, FKBP Prolyl Isomerase 10 (FKBP10), Regucalcin and Sulfite oxidase (SUOX)] (**Figure 3B**; **Table S4L**). Moreover, haptoglobin was shared in 100% of the tolerance publications, while MAP1 was present in 75% of the studies and other four proteins (APOE, C3, Ceruloplasmin and GC) appeared in 50% of the studies (**Figure 3C**; **Table S4M**). Moreover, 40 proteins (8.39%) were shared between IRI and rejection studies (**Table S4N**), 13 (2.73%) between IRI and tolerance (**Table S4O**), and 8 (1.63%) between rejection and tolerance (**Table S4P**) studies. A total of 1.05% of the proteins described (albumin, APOA1, C3, GSTA1 and hemopexin) were shared among the three different study types (**Figure 3D**; **Table S4Q**).

**Figure 4:**
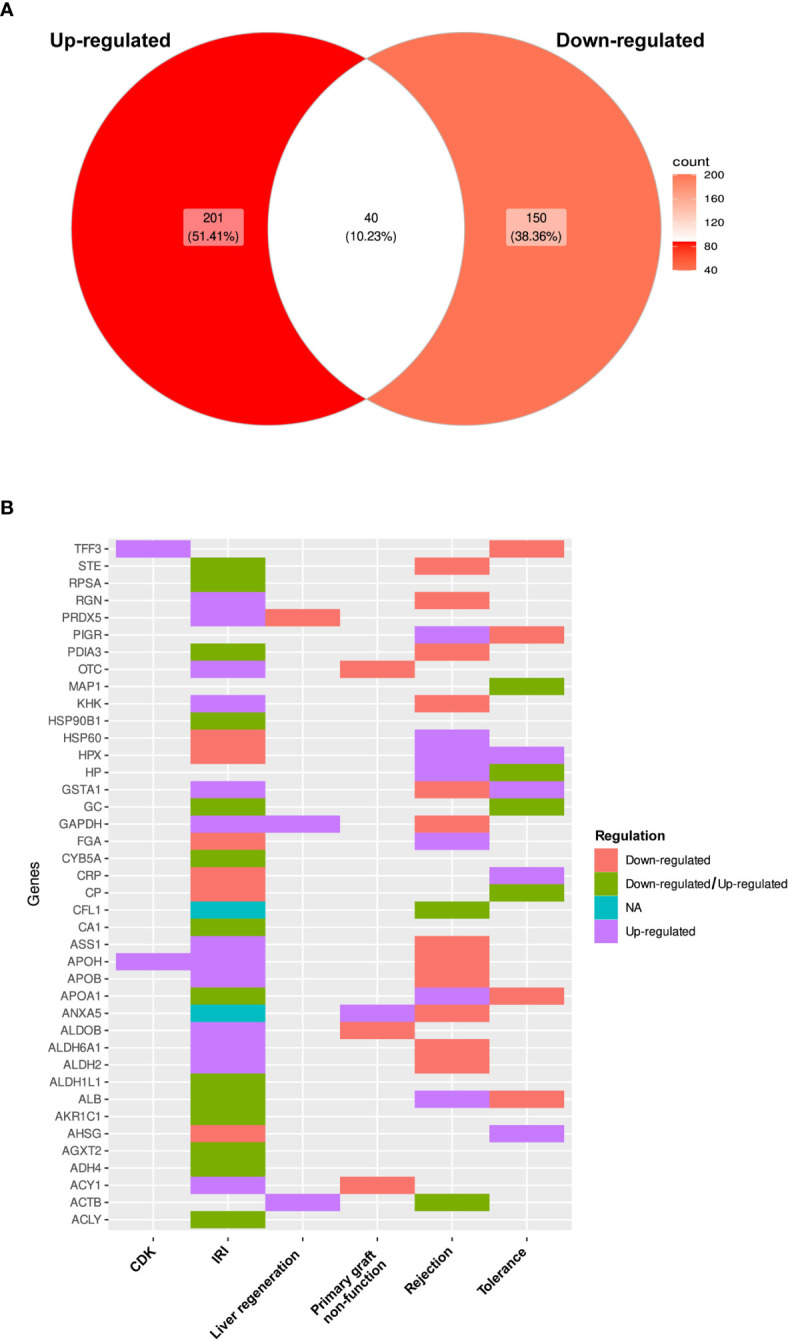
Differential pattern of protein expression. **(A)** Comparison of differential expression patterns observed for proteins among all selected studies expressed as a Venn diagram. **(B)** Heat plot of the list of proteins that had evidence supporting both types of expression changes.

Differential patterns of protein expression were reported for 78.67% of the listed proteins, in which 241 proteins were up-regulated among all study types or sample sources and 190 were down-regulated. Furthermore, 40 of these proteins (10.23%) were described as both up- and down-regulated, in several publications (**Figure 4A**; **Table S4R**). When analyzed by study type, IRI showed the most duplicated patterns [14 proteins including Estrogen sulfotransferase (STE), Ribosomal protein SA (RPSA), Protein disulfide isomerase family A member 3 (PDIA3), Heat shock protein 90 alpha family class A member 1 (HSP90B1), GC, Cytochrome B5 type A (CYB5A), CA1, APOA1, Aldehyde dehydrogenase 1 family member L (ALDH1L1), Albumin, Aldo-keto reductase family member C1 (AKR1C1), Alanine-glyoxylate aminotransferase 2 (AGXT2), Alcohol dehydrogenase 4 (ADH4) and ATP citrate lyase (ACLY)], followed by tolerance studies with four proteins (MAP1, Haptoglobin, GC and Ceruloplasmin) and graft rejection with two proteins (CA1 and ACTB) (**Figure 4B**).

Only Huang et al. (36) carried out a correlation analysis of proteomic results with transcriptomic data.

### Enriched Pathways

In this study, 47 and 43 pathways were associated, respectively, with the protein list for serum or liver samples. Metabolic pathways were the most representative in both cases, including carbon metabolism (hsa01200), glycolysis/gluconeogenesis (hsa00010), and amino acid biosynthesis (hsa01230) (**Tables S5A, B**). Although 70% of the pathways were shared (**Table S5C**), complement/coagulation cascades (hsa04610), and interleukin (IL)-17 signaling pathway (path04657) were specific for serum, whereas protein processing in the endoplasmic reticulum (hsa04141) was related exclusively to liver tissue samples. Analysis of specific studies showed that metabolic pathways (hsa01100) and complement/coagulation cascades were the principal pathways involved in IRI (**Table S5D**). Likewise, metabolic pathways were the most important in rejection (**Table S5E**), whereas cytokine-cytokine receptor interaction (hsa04060), complement/coagulation cascades, or IL-17 (path04657) and tumor necrosis factor (TFN) signaling pathways (path04668) were the most prominent in tolerance (**Table S5F**).

### Quality Evaluation

**Table S2** shows the methodological quality scores of the included studies according to the Downs and Black methods. The average score was 56.99%, and eight of the 22 studies achieved scores equal to or greater than 60%. In general, the studies clearly stated their objectives, hypotheses, and main findings. However, most of the studies did not properly describe the main confounders. Moreover, the number of participants was reduced in general, so the score in the representative source population and outcome measures was also low.

## Discussion

Proteome studies are powerful tools in translational clinical research. However, this relatively new and expensive technique requires a complex infrastructure (39) which might explain why there have only been a few studies and limited work focused in LT and why most have been conducted in the US and China during the last 15 years. Ideally, the techniques applied should be able to identify and quantify all proteins present in any clinical sample. To achieve this goal, it is essential to upgrade protein and peptide separation techniques, mass spectrometry instrumentation, and bioinformatics software together with the accession to complete sequence genome databases (40, 41). Unfortunately, deep proteome analysis is hampered by the high dynamic range in protein abundances (42) of an estimated seven orders of magnitude, while current mass spectrometry instruments can only analyze four orders of magnitude. Therefore, mass spectrometry-based proteomics requires the combination of different separation techniques to resolve complex mixtures of proteins to reach the goal of identifying and quantifying all proteins. 2D gels have been widely used since the first proteomics studies (43, 44), although they have been replaced by bottom-up or shotgun proteomics due to the increased number of proteins detected, reduced sample handling, and higher sampling throughput. The shotgun strategies are based on protease digestion of protein mixtures, peptide separation by liquid chromatography, and the identification by MS/MS of some tryptic peptides of each protein, covering a low percentage of their sequence, but allowing their identification (45). Furthermore, the stable isotope labeling of peptides (iTRAQ or TMT-tags) enables the quantification of the changes in abundance for thousands of proteins in a single experiment by multiplexing up to eleven different peptide samples (46, 47). Despite this yield, shotgun proteomics experiments can only detect half of the proteins present in a proteome (42) and the information obtained is limited to the few tryptic peptides that allow the identification of the protein. In contrast to 2D-gel-based techniques, we cannot distinguish between the different isoforms of a certain protein or if any of them have changed their concentration after LT. In clinical research, the high dynamic range in plasma protein levels is well known (48) for which several strategies have been developed to address this obstacle. Chromatographic methods for depleting the most abundant proteins present in serum/plasma (49) or the application of combinatorial hexapeptide ligand libraries (ProteoMiner™ beads) (50) enable the identification of hundreds of proteins using a shotgun proteomics approach (24, 34). However, the identification of thousands of proteins requires an extensive fractionation of serum/plasma using affinity and/or ionic chromatography before applying mass spectrometry-based proteomics to each fraction (26, 37). In 2D gel proteomics, protein quantification is performed using staining methods before protein digestion and mass spectrometry analysis. Modern sensitive techniques such as difference gel electrophoresis (DIGE) (51) or silver (52) staining protocols allow the detection of hundreds of protein spots. Furthermore, the design of DIGE experiments allows a reduced number of biological replicates compared to other staining methods such as silver or Coomassie Blue staining (53). However, 2D gel spot identification of hundreds of proteins hampers the proper quantification of each protein present in the spot using only the gel staining step (54). The presence of several proteins in the same spot also complicates their identification using Peptide Mass Fingerprinting (18, 19), a common mass spectrometry analysis method linked to 2D gel proteomics (55); thus, MS/MS is required to improve protein identification (56). The proteins identified by both 2D gel and shotgun proteomics approaches require validation using other methodologies since they are inferred from peptides sequenced by MS/MS and not from complete protein. In contrast to bottom-up proteomics strategies that involve protease digestion before mass spectrometry analysis, top-down proteomics strategies identify and quantify intact proteins directly through their fragmentation in the mass spectrometer (57). This methodology enables the study of individual proteoforms (58), which is not possible through “bottom-up” proteomics strategies, opening a new approach in clinical proteomics. Therefore, top-down proteomics can describe protein species generated due to RNA splicing, post-translational modification, or proteolysis. However, several obstacles still hamper complete proteome analysis, such as the proteoform pre-fractionation step and the limitation of high-resolution mass spectrometers. The application of gel-eluted liquid fraction entrapment electrophoresis (59), as a proteoform pre-fractionation method, in addition to high-resolution mass spectrometry, has allowed the identification of proteoforms that describe novel protein biomarkers associated with the liver transplant rejection (32). Another approach to solving the difficulties associated with the high dynamic range present in biological samples is to apply targeted proteomic tools as antibody arrays. This technology allows the quantification of hundreds of proteins previously selected due to their clinical interest. This approach has been applied in the field of LT to identify liver biomarkers using hepatic biopsies (20) or plasma (25, 30). The development of equipment combining fluorescence and multiplexing technologies has allowed a notable increase in the numbers of proteins detected in small volumes of sample [189 proteins (25, 30) compared to 40 for antibody arrays (20)].

Rat LT is a well-established experimental model (60) with hundreds of publications in the last 15 years. Moreover, preclinical studies with rodents have been accepted as a previous step in clinical trials (61). However, only 14% of the proteins found to be differentially expressed in rat studies were replicated in human samples. Although these species share 95% of their genes (62), there are differences in their liver transcriptomes (63). Similarly, hepatocytes synthesize most of serum proteins in the endoplasmic reticulum (ER), so that serum levels of hepatocyte-made proteins comprise significant biomarkers that reflect both systemic processes and liver status (64). However, only 2% of proteins were shared between serum and liver tissue in our SLR. This might be biased by the fact that the tolerance studies always started from serum, whereas IRI and graft rejection studies started mainly with biopsies. Nevertheless, the processes that took place in both samples implied similar enrichment of pathways but included different proteins. Moreover, protein processing in the ER pathway appeared enriched only in liver tissue samples. However, the presence of shared proteins between liver tissue and preservation solution samples increased up to 14%, as many cellular components are released following damage occurring during cold ischemia (65). It is, therefore, not surprising that both samples showed metabolic pathways enrichment, as liver is known as the metabolic factory (66) (**Table S5G**).

Ischemia-reperfusion is a pathological condition resulting from an initial restriction of blood supply to an organ followed by the restoration of perfusion and reoxygenation (67). Although surgical techniques and immunosuppressants increase transplant success rates, allograft rejection remains a major problem, with a maximum incidence within the first weeks (68). IRI after deceased donor transplantation increases the rate of acute and chronic rejection and is estimated to account for 10% of early organ failure (69). Thus, it is not surprising that IRI and rejection studies shared more proteins. However, 13 of these proteins appeared indistinctly up- or down-regulated in both study. Likewise, 17 proteins could not be compared since they were identified in an IRI study conducted with preservation fluid (35), where the differential expression pattern was not available. In other words, only 25% of the proteins shared between IRI and rejection studies showed the same patterns. Nevertheless, reperfusion injury, results not only from metabolic disturbances but also from an inflammatory immune response (70). However, although there is an enrichment of different metabolic pathways in both IRI and rejection, only the related immune response complement/coagulation cascades and hypoxia inducible factor 1-alpha (HIF-1a) signaling pathways appear to be somewhat prominent in IRI and rejection samples, respectively. Apoptosis plays an important role in IRI during LT. HIF-1a may trigger liver apoptosis following IRI through the induction of hypoxically regulated genes (71).

Operational tolerance is defined as the state in which the allograft is allowed to survive at a level that provides adequate clinical function in the absence of IS (72). The achievement of immune tolerance to an allogeneic graft is a field of intense research, fueled by a critical need to avoid IS-related side effects (73). Different groups have identified biomarkers to distinguish tolerant patients from those who are going to experience rejection, including genes (74, 75), T cell receptors (76–78), activated regulatory T-cells (Treg) and related miRNAs (79). However, proteomic analysis has not been commonly used to study tolerance. The Chen group in Kaohsiung Chang Gung Memorial Hospital (Taiwan) has studied the proteomic signature of spontaneous tolerance in a rat model of LT (17, 23, 33). The rat models of tolerance can be induced spontaneously in LT between different strains (80). In this way, studies in tolerance proteomics were more similar, with haptoglobin reported to be differentially expressed in every publication. Same authors showed haptoglobin to be differentially expressed in serum from a liver transplant recipient after IS withdrawal (81). Moreover, in their proteomic study using Luminex Bead technology, Levitsky et al. (30) reported haptoglobin as significantly differentially expressed. However, haptoglobin is reported to be both down- (23) and up-regulated (17, 33) in different studies by the same authors. Moreover, haptoglobin was also reported in one rejection study (21), when tolerance is a totally antagonistic phenomenon. This protein has also been identified as among the 12 most abundant plasma proteins in serum (38).

Proteomics is a promising technique that can be used to detect a large number of proteins. Even so, progress remains slow and further research is needed to develop a treatment (82). The experimental variability between different study types and the low numbers of participants do not help to identify specific biomarkers for IRI, rejection, or tolerance. Increasing the number of samples by means of cheaper validation methods is a likely possibility. Similarly, there is an urgent need to eliminate the most abundant proteins when working with serum or plasma samples. Selective depletion of a dozen high-abundance proteins extends analyses down about 1–2 orders of magnitude and depletion of about 150 major proteins extends the range to approximately 10,00-fold lower concentrations (38).

Although proteomics in LT, or any other field, is extremely promising on its own, the future of this technique lies in the improvement of the identification of proteoforms and their integration with other techniques such as genomics and metabolomics, which will help to provide a global view of the interactome of all the agents involved in a particular biological process.

## Data Availability Statement

The original contributions presented in the study are included in the article/**Supplementary Material**. Further inquiries can be directed to the corresponding author.

## Author Contributions

PR, FS-B, JP, RR-C, and AB-M participated in de design of the study. VL-L, JM-A, FP-S, CT-M, and AB-M participated in the performance of the research. RR-C, VL-L, JM-A, and AB-M participated in data analysis. All authors contributed to the article and approved the submitted version.

## Funding

AB-M was funded by Fundación Mutua Madrileña, Grant/Award Number: AP171362019 and Instituto de Salud Carlos III, Grant/Award Number: PI20/00185. JP and RR-C were funded by Instituto de Salud Carlos III, Grant/Award Numbers: PI17/00489 and PI13/02870, respectively. Funding sources provided financial support but had no involvement in study design, collection, analysis and interpretation of data.

## Conflict of Interest

The authors declare that the research was conducted in the absence of any commercial or financial relationships that could be construed as a potential conflict of interest.

## Publisher’s Note

All claims expressed in this article are solely those of the authors and do not necessarily represent those of their affiliated organizations, or those of the publisher, the editors and the reviewers. Any product that may be evaluated in this article, or claim that may be made by its manufacturer, is not guaranteed or endorsed by the publisher.

## Abbreviations

ACTB, Actin beta; APO, Apolipoprotein; CA1, Carbonic anhydrase 1; CKD, Chronic kidney disease; C3, Complement C3; DIGE, Difference gel electrophoresis; ER, Endoplasmic reticulum; GC, GC vitamin D binding protein; GSTA1, Glutathione S-transferase alpha 1; IS, Immunosuppression; IL-17, Interleukin-17; IRI, Ischemia reperfusion injury; iTRAQ, Isobaric tags for relative and absolute quantitation; LT, Liver transplantation; MAP1, Microtubule associated protein 1; PRISMA, Preferred Reporting Items for Systematic Reviews and Meta-Analyses; SLR, Systematic literature review; MS/MS, Tandem mass spectrometry; 2D gel, Two-dimensional gel electrophoresis.
