# Supplementary material for: Proteomics in Liver Transplantation: A Systematic Review
Source: Front Immunol. 2021 Jul 26;12:672829. doi: 10.3389/fimmu.2021.672829 (PMC8350337; doi:10.3389/fimmu.2021.672829)
Supplement: Supplementary file 1 [file Table_1.docx]

**Table S1.** Specific searches performed in the three databases evaluated for the SLR.

| **Database** | **Search term** |
| --- | --- |
| Scopus | [TITLE-ABS ( ( liver  AND  transplantation  OR  liver  AND transplant  OR  liver _AND graft  OR  liver  AND allograft )  AND  ( proteomics  OR  proteome ) )](https://www.scopus.com/results/documentSpellSuggest.uri?sort=plf-f&src=s&sid=9445dc0667a04a6615bf751ed6d8cc3f&sot=a&sdt=a&sl=128&s=TITLE-ABS+%28+%28+liver+AND+transplantation+OR+liver+transplant+OR+liver+graft+OR+liver+allograft+%29+AND+%28+proteomics+OR+proteome+%29+%29&origin=resultslist) |
| PubMed | (((((liver transplantation[Title/Abstract]) OR (liver graft[Title/Abstract])) OR (liver transplant[Title/Abstract])) OR (liver allograft[Title/Abstract])) AND (proteomics[Title/Abstract] OR (proteome[Title/Abstract])) |
| Science Direct | (((((liver transplantation[Title/Abstract]) OR (liver graft[Title/Abstract])) OR (liver transplant[Title/Abstract])) OR (liver allograft[Title/Abstract])) AND (proteomics[Title/Abstract] OR (proteome[Title/Abstract])) |
